# Thank you to *eClinical Medicine’s* clinical and statistical peer reviewers in 2023

**DOI:** 10.1016/j.eclinm.2024.102460

**Published:** 2024-01-24

Raveendran A V

Winston Abara

Guilia Abate

Kaja Abbas

Ebrahim Abbasi

Penelope Abbott

Hazreen Abdul Abdul Majid

Baharudin Abdullah

Hairil Abdullah

Haim Abenhaim

Martha Abshire Saylor

Deepak Acharya

Lisa Adams

Florence Ader

Jeremy Adler

Sam Afkhami

Anil Agarwal

Alvar Agusti

Anas Ahmed

Kiwamu Akagi

Seun Akeju

Karolina Akinosoglou

Onno Akkerman

Jason Akulian

Jennifer Albrecht

Kefyalew Alene

Annet Ngabirano Alenyo

Kristine Allin

Amin Alousi

Hanny Al-Samkari

Margit Alt Murphy

Lauren Alvis

Nisreen Alwan

Bahman Amani

Hanna Åmark

Namasivayam Ambalavanan

Edward Kwabena Ameyaw

Stephanie A Amiel

Jenerius Aminawung

Eitan Amir

Lars Andersen

Işıl Işik Andsoy

Olivia Aranha

Andrew Argent

Jean Benoît Arlet

Pedro Armario

Alessandro Armuzzi

Sidse Arnfred

Doron Aronson

Nidal A Asaad

Takashi Asai

Adam Konrad Asghar

Diane A I Ashiru-Oredope

Breton Asken

Stephen Atkin

Raja Atreya

Sylvain Audia

Karen Austrian

Sergey Avdeev

Anthony M Avellino

Francisco Ayala

Paul T Y Ayuk

Sina Azadnajafabad

Hassan Aziz

Kengo Azushima

Peter Baade

Ana Babic

Suma Babu

Brian Badgwell

Kamran Badizadegan

Luis Bahamondes

Shasha Bai

Xue Bai

Stephen Bain

Olusegun Baiyewu

Tamam Bakchoul

Yasin Hasan Balcioglu

Alexandre Pazetto Balsanelli

Hideaki Bando

Giuseppe Luigi Banna

Heike Bantel

Yanping Bao

Peter Baptista

Vickie Baracos

Stefano Barco

Shaun Barnabas

Alfred Sidney Barritt

Aluisio Barros

Melissa C Bartick

Alfred Bartolucci

Ran Barzilay

Buddha Basnyat

Quique Bassat

Rajit K Basu

Somprakas Basu

Robert J Battat

Valentin Bauer

Yvonne Baumer

Iacopo Baussano

Nuray Bayar Muluk

Jagadeesh Bayry

Eliza Beal

Alessandra Bearz

Kristen M Beavers

Karl Bechter

Prakash Behere

Abdelouahab Bellou

Bjørn Bendz

Elinor Ben-Menachem

Ilies Benotmane

Agathe Béranger

Alexander E Berezin

Ivan Berlin

Inge Bernstein

Andrea Bettinelli

Woldesellassie Mequanint Bezabhe

Sanjay Bhagani

Gayatri Bhatia

Rohit Bhatia

Merijn Bijlsma

Thomas Bilterys

Giuseppe Biondi-Zoccai

Gallus Bischof

Therese Bittermann

Richard Blanch

Daniel Blatt

Mikaela Bloomberg

Daniel Blumberger

Jason Boardman

David Boettiger

Magdalena Bofill

Giorgio Bogani

Pawel Bogdański

Niccolo Bolli

Pierre-Adrien Bolze

Riccardo Bonfanti

Stefan Bornstein

Andrea Borondy Kitts

Domenico Bosco

Rita Bottino

Malika Bouhenia

Liza Bowen

Eileen Boyle

Kayvan Bozorgmehr

Westyn Branch-Elliman

Valerie Brandt

Mats Brannstrom

Jeremy Braybrooke

Lorraine Brennan

Hermann Brenner

Keno Bressem

Stéphanie Breukink

Francesco Brigo

Fernando Bril

Meredith Brooks

Matthijs Brouwer

Willem Pieter Brouwer

Elizabeth Brown

Gary P Brown

Jamie Brown

Cheryl L Brunelle

Oliviero Bruni

Francesco Bruno

Richard Bryant

Anna Brytek-Matera

Adam H Buchanan

Joshua Buckman

Donal J Buggy

Christoph Bührer

Danilo Buonsenso

Johan Burisch

Rachael Burke

James Bussel

Joanne E Butterworth

Sara Buttery

Christopher Byrne

Guoxiang Cai

Lilian Calderon-Garciduenas

Patrina Caldwell

Denton Callander

Andrea Calvo

Jose Antonio Cañas

Guilherme Grossi Lopes Canca̧do

Christopher Cannon

Feng Cao

Gabriel Capella

Simon Capewell

Gabriella Captur

Augusto Caraceni

Cheryl Carcel

Mariana Carmen Chifiriuc

Suzan Carmichael

Rodrigo Carrillo-Larco

Ben Carter

Rosario Caruso

Fidelia Cascini

Desiree Caselli

Jonathan Casey

Peter Cashin

Antonella Castagna

Jessica Catchpole

Cyrielle Caussy

Maurizio Cecconi

Leo Anthony G Celi

Claudio Cerchione

Karim Chamari

Nola Chambers

Sherwin Chan

Shirley Chiu Wai Chan

Chinkuo Chang

Junbiao Chang

Kiara Chang

Ling-sai Chang

Keith Chappell

Salome Charalambous

Phunchai Charatcharoenwitthaya

Nathalie Charpak

Sanjay Chawla

Bin Chen

Eric Chen

Hongda Chen

Jiayi Chen

Jie Chen

Kai Chen

Qiang Chen

Tianmu Chen

Xiaoyuan Chen

Joyce Y Cheng

Xin Cheng

Ying Cheng

Ka Shing Cheung

Chunru Chien

Natsayi Chimbindi

Fumiko Chino

Mohammod Jobayer Chisti

Virasakdi Chongsuvivatwong

Janejit Choovuthayakorn

Supriya Chopra

Rok Seon Choung

Elaine Chow

Anne Vinggaard Christensen

Chong Chyn Chua

Melvin Chua

Dmytro Chumachenko

Felix Chun

Helen Chun

Kelly Chun

Hyun Cheol Chung

Gavin J Churchyard

Fortunato Ciardiello

Andrea Cipriani

Antonio Cittadini

Una Clancy

Olivier Claris

Ralf Clemens

Noa Cleveland

Benjamin Click

Gary Clifford

Tammy Clifford

Cheryl Cohen

Raffaella Colombatti

Simon Conroy

Jean-Michel Constantin

Andrew Copas

Rosie Cornish

Samuele Cortese

Chandler Cortina

Elisabetta Costantini

Menino Cotta

Elizabeth Cotter

Gillian Coughlan

Keith Couper

Luciano Coutinho

Allan Covens

Frances Cowan

Julia Cozart

Theodore Cross

Wiesław Cubała

Alessandro Cucchetti

Longzhu Cui

Sarah Cumming

Vasa Curcin

Giuseppe Curigliano

William C Cushman

Stefania Cuzzubbo

Leticia Czepielewski

Andrea Da Porto

Omid Dadras

Haijiang Dai

Hiroyuki Daida

Farouk Dako

Paulo Tarso Dalcin

David D'Alessandro

Stephane Dalle

Lia D'Ambrosio

Jun Dang

Maria Giovanna Danieli

Leon Danon

Jamel Daoud

Gary Darmstadt

Paramita Dasgupta

Andrew Dauber

Lori L Davis

Eran Dayan

Mark Dayer

Nanne de Boer

Sara De Carolis

Angharad N de Cates

Roselle de Guzman

Christelle De La Fouchardiere

Vincent de Parades

Livia De Picker

Fátima P de Souza

Rory de Vries

Saskia de Wildt

Charles DeCarli

Desh Deepak

Giuseppe Defeudis

Sharon Dekel

Francesca Del Bufalo

Lucia Del Mastro

Constance Delaugerre

Artur Figueiredo Delgado

Julio Delgado

Gerco den Hartog

Dirk den Ouden

Lynette Denny

Luc J J Derijks

François Destrempes

Haryana Dhillon

Sameer Dhingra

Massimo Di Maio

Giulia Di Stefano

Gian Luca Di Tanna

Christoph Frank Dietrich

Jennifer Dietrich

Subba Digumarthy

Amanda Dimachkie Nunnally

George Dimopoulos

Andrew DiNardo

James DiNardo

Tao Ding

Yingying Ding

Jacqueline Dinnes

Nicola Disma

Oliver Distler

Priyanka Dixit

Matthew Dodd

Peter Dodd

Mangat Dogra

Michael J Dohopolski

Daniele Dona

Yujun Dong

Sabrina Donzelli

Gregory Dore

Johannes Dorst

Tonia Douglas

Robert Kokou Dowou

David Doyle

Peter Dreger

Karen Drukker

Dongping Du

Mengxi Du

Mulong Du

Yuncheng Du

Emmanuelle Duceppe

Simon Ducharme

Michel P Ducreux

Marieke Duiverman

Reinhard Dummer

Tim Duong

Nicolas Dupin

Dmitry V Duplyakov

Jibran Durrani

Matthew Durstenfeld

Laura Dwyer-Lindgren

Mariusz Dyląg

Shawn Eagle

Ramin Ebrahimi

Linda Oneal Eckert

Julien Edeline

Fabio Efficace

Bijan Eghtesad

Susumu Eguchi

Harald Ehrhardt

Aristides G Eliopoulos

Sascha Ellington

Marta Elliott

David Ellwood

Shaker El-Sappagh

Mohammad Abdalla Eltahlawi

Mark Emberton

Monika Engelhardt

Michiel Elardus Erasmus

Masahiro Eriguchi

Carlos Escobar Cervantes

Mohammed Eslam

Michelle Estrella

Paolo Eusebi

Laura Evangelista

Chukwuka Eze

Chiara Fabbri

Francesco Facchinetti

Gian Paolo Fadini

Akl Fahed

Alexandre Faisal-Cury

Zuyi Fang

Eric Faragher

Mohammad Farahpour

Aline Fares

Andrew Farmer

Jennifer Farrar

Michelle Farrar

Forough Farrokhyar

Nadia Fathallah

Bruno Fautrel

Seena Fazel

Yunyun Fei

Judith Feinberg

Jordan Feld

Ursula Felderhoff-Müser

Eleonora Feletto

Jiexiong Feng

Elisbeth Fernell

Marc Ferrante

Clarissa Ferrari

Ana Ferreiro

Estêvão Figueiredo

Carsten Finke

David Finkelhor

Josef H Finsterer

Alexandra Fisher

Patricia Fitzpatrick

John Flack

Alastair Flint

Stuart Flint

Zachariah Foda

H Rui Martins Fonseca

Roberta Forlano

Fabio Formaglio

Emil Loldrup Fosbøl

Maria Pia Foschino Barbaro

James Fotheringham

Lauren A Fowler

Nora Franceschini

Massimo Franchini

Orla M Franklin

Tom Freeman

Corinne Frere

Florian Freudenberg

Carsten Friedrich

Lisa Frigati

Antoine Froidure

Nikolaj Frost

Xinxing Fu

Akihiro Fujino

Mitsuhiro Fukata

Nancy Fullman

Toshi A Furukawa

Junji Furuse

William Fusco

Eli Gabbay

Shi-Rui Gan

Afschin Gandjour

Sumanth Gandra

QingLei Gao

Xiang Gao

Christopher Gast

Nick Gebruers

Franco Gemignani

Pietro Gentile

Biju George

Jacob George

Marco Geraci

Tamer Gheita

Ehsan Ghorani

Uday Ghoshal

Diana Giannarelli

Nicolae Gică

Judy Wawira Gichoya

Clare Gilbert

Luis Andrés Gimeno-Feliu

Eva Giné

Corrado Girmenia

Jacopo Giuliani

Melissa Gladstone

Nancy Glass

Kristen M Glenister

Evgin Goceri

Mariette Goddijn

Catherine Godfrey

En Lin Goh

Ralf Gold

David Gomez-Almaguer

Fernando Gomollon

Wenping Gong

Tomas Jose Gonzalez-Lopez

José Luis Górriz

Jiangtao Gou

Radjiv Goulabchand

Asger Granfeldt

Alessandro Granito

Lorraine Greaves

Peter Wagoner Greenwald

John Gregson

Gorm Greisen

Jörn Grensemann

Jens Greve

Paul B Grogan

Francesco Grossi

Axel Grothey

Ruth E Grunau

Jionghui Gu

Stefano Guerra

Pablo Guisado Vasco

Martin Gulliford

Jan Gunst

John Gunstad

Gunar Günther

Yutao Guo

Rajeev Gupta

Samir Gupta

Ankur Gupta-Wright

Eero Haapala

Mohamad Habes

Walid Habre

Ruth A Hackett

Samy Hadjadj

Sezaneh Haghpanah

Yong Hai

Shahab Hajibandeh

Thorvardur Halfdanarson

Wayne Hall

Kevin Hallgren

Yohhei Hamada

Mehdi Hamadani

Mika Hamer

Guang Han

Ying Han

Shinsuke Hanatani

Dong Hang

Katrine Hansen

Tom Hansen

Michael Harhay

Christoph Härtel

Kiyoshi Hasegawa

Kenji Hashimoto

Cesare Hassan

Jessica Hassel

Hamid Hassen

Satoshi Hattori

Steen Haugaard

Sarah Hawkes

Mary S Hayney

Daihai He

Yukai He

Yulei He

Andras Heczey

Darren Hedley

Axel Heidenreich

Markku Heikinheimo

Yoram Hemo

Lizza Hendriks

Julie Hennegan

Alex Herrera

Ken Herrmann

Chaim Hershko

Morten Hesse

Gerd Heusch

Robert Heyderman

Alisa Higgins

Allan Hildesheim

Aileen Hill

Andrew Hill

Briony Hill

Loreena Michelle Hill

Jens Hillingsø

Robert Hinch

Carsten Hjorthøj

Prahlad Ho

John Hodsoll

Kara Holloway-Kew

Trygve Holmøy

Caroline Homer

Sung-Jin Hong

Samar Kumar Hore

Nir Horesh

Benjamin Horne

Christopher Horvat

Angela Horvath

Erzsébet Horváth-Puhó

Markus Hovd

Heather Hower

Jennifer Hoy

Htet Lin Htun

Yali Hu

Yongxian Hu

Zhongjie Hu

Jia Huang

Xin Huang

Yi-Hsiang Huang

Allyson S Hughes

Jacques Hugon

Henkjan Jan J Huisman

Keith Humphreys

Clara Humpston

Ivan F N Hung

Mathilde M Husky

Bing-Fang Hwang

Natalie Hyde

Pirro Hysi

Massimo Iacoviello

Gianluca Ianiro

Katsuro Ichimasa

Makoto Ide

Masafumi Ihara

Andreas Ihle

Mark Iles

Jorma Ilonen

Arun Inamadar

Hamish Innes

Ana-Maria Iosif

Kenneth Iregbu

Shizukiyo Ishikawa

Toru Ishikawa

Md Mazharul Islam

Md Monirul Islam

Khalida Ismail

Masaya Ito

Yasser Iturria-Medina

Abbygail Jaccard

Jeremy Jacobs

Henning Jacobsen

Preetesh Jain

Guruprasad Jambaulikar

Tukur Jamilu

Naveed Janjua

Megan Jarman

Dona M P Jayakody

Martina J Jelley

Kristina Jenei

Jens Richardt Møllegaard Jepsen

Nicholas Jewell

Rachel Jewkes

Peng Jia

Gening Jiang

Heng Jiang

Yu Jiang

Hui Min Jin

Ketao Jin

Zhichao Jin

Youngji Jo

Esaú João

Douglas Johnson

Nicholas Johnson

Laimas Virginijus Jonaitis

Michael Jones

Wutong Ju

Manel Juan

Steven Julious

Christian Jung

Dong-hwan Jung

Melissa Kacena

Antonia Kaczkurkin

Hiroshi Kagamu

Leila C Kahwati

Angela Kaindl

Emma Kalk

Rahul Kalla

Manali Kamdar

Lars Kamolz

Malek Kamoun

Jolly Kamugisha

Yoshinobu Kanda

Tadashi Kaneko

Sung-Yoon Kang

Ahmad Kantar

Suman Kanungo

Gilaad Kaplan

Peeter Karihtala

David Karsenti

Naoki Kashihara

Ken Kato

Antonis Kattamis

Alexander Kautzky

Roop Kaw

Takumi Kawaguchi

Yoshiharu Kawaguchi

Fengfeng Ke

Karen Keddy

Fahrettin Keleştemur

Daniel Kelly

Mark Kelson

Zoe Kelson

David Kennaway

Nanda Kerkar

Mohammad Kermansaravi

Winfried Kern

Stephen Kerr

Shelli Kesler

Bart Keymeulen

Puja Khaitan

Ahsan Khandoker

Ali Khashan

Sajad Khazal

Zeinab Khodaverdi

Adree Khondker

Sepideh Khoshnevis

Rajat Khosla

Jérémy Khouani

Dinesh Khullar

Biniam Kidane

Hae-Young Kim

Ho Cheol Kim

Jae Hui Kim

Jung-Sun Kim

Nalee Kim

SeungUP Kim

Dorien Kimenai

Tania King

Ryo Kinoshita

Amit K Kishore

Harald Kittler

Eric Klein

Christoph Kleinschnitz

Michael Klompas

William C Knowler

Rebecca Knowles

Inga Koerte

Fumitaka Koga

Hirofumi Kogure

Masahiro Kohzuki

Issei Komuro

Evangelos Kontopantelis

Neha Korde

Keisei Kosaki

Heikki Koskela

Konstantinos C Koskinas

Suresh Kotagal

Tomomi Kotani

Kou Kou

Stjepana Kovac

Andreas Kremer

James Krinsley

Rathika Krishnasamy

Rahul Krishnatry

Florian Kronenberg

Makoto Kudo

Edward Kuijper

Ilari Kuitunen

Ravina Kullar

Jonna Kuntsi

Serhan Küpeli

Kalle Kurppa

Tatyana Kushner

Oliver Kuß

Vivek Kute

M Tezer Kutluk

Hmwe Kyu

Carlo La Vecchia

Laura Labriola

Kathleen E Lacy

Chih-Cheng Lai

Quirino Lai

Péter László Lakatos

Sham Lal

Fiona Lalloo

Jack Lambert

Marcello Lanari

Simona Lattanzi

Eric Lau

Alexis Laurent

Carl Lavie

Federico Lavorini

Jeffrey Lazarus

Nguyen Quoc Khanh Le

Xiuning Le

Alexandre Leclercq

Jae Seung Lee

Maria Lee

Seung-pyo Lee

Tsair Fwu Lee

Way Seah Lee

Yong-ho Lee

Heather E Leeper

Rachel Lees

Vicky Lehmann

Daniel Leightley

Anja Leist

Philipp Lenz

Andrea Lenzi

Marc Leone

Stuart Leske

Nader Lessan

Noémie Letellier

Helena Lewis-Smith

Cheng-Ta Li

Dongdong Li

Huiping Li

Jian Li

Jingxin Li

Mian Li

Molly Li

Ni Li

Ning Li

Sheyu Li

Yan Li

Yiran Li

Zhengyan Li

Wei-Chih Liao

Yanhui Liao

Silvan Licher

Howard John Lim

Young-Suk Lim

Chenyuan Lin

Chinsheng Lin

Haotian Lin

Ro-Ting Lin

Shih-Hua Lin

Lauri Linder

Anthea Lindquist

Outi Linnaranta

Miklos Lipcsey

Gerald Lipshutz

Chen Liu

Chunfeng Liu

Hanruo Liu

Junfeng liu

Qin Liu

Tian Liu

Tong Liu

Xiaoqin Liu

Zhenyu Liu

Per Ljungman

Marci Lobel

João Lobo

Franco Locatelli

Marie Löf

Erik Torbjørn Løhre

Ansgar Wilhelm Lohse

Raúl López-Izquierdo

Florian Lordick

Giuseppe Losurdo

Olabisi Morebise Loto

Sharon Love

Maria L Lozano

Jun Lu

Lin Lu

Yuanyuan Lu

Alejandro Lucia

Hsiang-Ning Luk

Tzu Tsun Luk

Susi Lund

Huiyan Luo

Peng Luo

Yuan Luo

Jay Lusk

Markus Luster

Miriam Lynn

Human M. Fatemi

Jennifer Ma

Ya'nan Ma

Joseph E Maakaron

Andrew Maas

Michiel Maas

Andrew Mackinnon

Peter MacPherson

Jagmeet Gurminder Madan

Sheri Madigan

Lola Madrid

Hideki Maeda

Fabrizio Maggi

Mathieu Maheu-Giroux

Hai-Qiang Mai

Govind Kumar Makharia

Polyzois Makras

Talía Malagón

Umberto Malapelle

Reza Malekzadeh

Amyn Malik

Domenico Mallardo

John A Maluccio

Mattias Mandorfer

Jayawant Mandrekar

Jessica Mandrioli

Nirav Maniar

Pinelopi Manousou

Aaron Mansfield

Kathryn Mansfield

Mohammad Ali Mansournia

Oriol Manuel

Anthony Muchai Manyara

Limin Mao

Sithokozile Maposa

Antonella Marangoni

Giovanni Marasco

Georgios Antonios Margonis

Joseph Marion

Niklas Marklund

Daniel Ruivo Marques

Michael T Marrone

Wanda Leigh Martin

Carmela Martinez

Pablo Martínez-Camblor

Javier Martinez-Trufero

Rosa María Martín-Mateos

Francisco Martín-Rodríguez

Paulo Ricardo Martins-Filho

Marta M Maslej

Takahiro Masuda

María-Victoria Mateos

Manu Mathur

Prashant Mathur

Jordi A Matias-Guiu

Rubeta Matin

Koji Matsuo

Claudia Matteucci

Sébastien Maury

Sophie I Mavrogeni

Anoop M Mayampurath

Stephen McCall

Eric McCollum

Brian McCrindle

Noel McElvaney

Patrick McGann

Amanda McIntyre

Hugh McMillan

Hayden McRobbie

Alberto Medina

Ushma C Mehta

Thomas Meinertz Dantoft

Asuncion Mejias

Maria Melchior

Nico Melzer

Gabriel E Mena

Nahum Mendez-Sanchez

Nicolas F Méneveau

Hongdao Meng

Xiangming Meng

Ellen Menkhorst

Giovanni Meola

Hamid Merchant

Ricarda Mewes

Tim Meyer

Laure Michel

Saskia Middeldorp

Philippa Middleton

Karsten Midtvedt

Filippo Migliorini

Takahiro Mihara

Tim Millar

Bruno Millet

Matthieu Million

Phillip Minar

Adrian Minson

Saori Mishima

Kimberly Mitchell

William Mitchell

John Mitrofanis

Kathleen F Mittendorf

Bettina Mittendorfer

Etsuko Miyagi

Pamela A Moalli

Vicent Modesto i Alapont

Richard Moffitt

Norliza Mohd Mohd Noor

Michelle Mollica

Elizabeth Molyneux

Paul Monach

Manuel Monreal

Bernhard Montag

Paolo Montaldo

Ali Montazeri

Giovanni Monteleone

Jacqueline Montes

Antonio Montresor

Daniel Morales

James P Moriarty

Steffen Moritz

Chigusa Morizane

Cécile Moro

Laust Hvas Mortensen

Kevin Mortimer

Lindsay M Morton

Elizabeth C Moser

Ralph Mösges

Julia Mueller

Tshilolo Muepu

Kim Mueser

Irfan Muhammad

Maria Lorenza Muiesan

Debabrata Mukherjee

Deborah Mukherji

Chris Jacob Johan Mulder

Christiaan Mulder

Fliss Murtagh

Srinivas Murthy

Venkatesh Murthy

Teemu Murtola

Baba Musa

Zia Ul Mustafa

V Raman Muthusamy

Emir M Muzurović

Paul Myles

Veit Mylius

Sharon A Nachman

Jason Nagata

Joshua Nagler

Harish Nair

Tateaki Naito

Tomoki Naoe

George E Naoum

Edmund Nartey

Yvonne Nartey

Mohammad Javad Nasiri

Avindra Nath

Jacob Nattermann

Sonya Negriff

Catherine Nelson-Piercy

Shamim Nemati

Yuriy Nesterko

Ursula Nestle

Gopalakrishnan Netuveli

Tryggve Nevèus

Riccardo Nevola

Robert Newton

Bart Neyns

Qin Xiang Ng

Phuong Nguyen

Emanuele Nicastri

Ole Haagen Nielsen

Suzanne Nielsen

Hendrik Niemarkt

Karen Nieuwenhuijsen

Anka Nieuwhof-Leppink

Els Jacqueline Maria Nieveen van Dijkum

Giel Nijpels

Hiroshi Nishihara

Tianye Niu

Esin Nkereuwem

Uche H Nnebe-Agumadu

Akihiro Nomura

Gill Norman

John Norrie

Seyed Mehdi Nouraie

Zoann Jane Nugent

Margaret O’Hara

Godfrey P Oakley

Ayodele Ogunleye

Jaehoon Oh

Kazunori Oishi

Naoko Okiyama

Özgür Öktem

Tsuyoshi Okura

Catherine Oldenburg

Émilie Olié

Catherine Olsen

Graziano Onder

Gabriel Oniscu

Bob Opoka

Pedro Ordunez

Walter Orenstein

Mark Orme

Andrés Ortiz

Esteban Ortiz-Prado

Sophie Orton

Lauren M Osborne

Asishana Osho

Johanna Ospel

Marlies Ostermann

Mattias Osti

Afshin Ostovar

Dylan O'Sullivan

Megan Othus

Wim Otte

Maria Otth

Ignatius Ou

Mehdi Oualha

Mamadou Ouattara

Carolyn Owen

Mayowa Owolabi

Neil Oxtoby

Yukio Ozaki

Stefano Palermi

Romain Palich

Vanessa Palzes

Daniel Pan

Harshita Pant

Jean-Jacques Parienti

Daniel Park

Haesuk Park

Pierre Parneix

Carlos Luís Parra-Calderón

Gianluigi Pasta

Shyam Patel

Nikhil Patkar

Sanjay Patole

Gary L Pattee

Lillian Pazvakawambwa

Maria Teresa Pellecchia

Adriaan Penson

Richard Penson

Pietro Pepe

Juan Pericas

Abhilash Perisetti

Francesco Perrone

Marcello Persico

Olumuyiwa James Peter

Sanne A E Peters

Jeff Zarp Petersen

Philip John Peyton

Elisa Pezzola

André Pfob

Marco Picardi

Maria Carmela Piccirillo

Lorenzo Piemonti

Fiammetta Piersigilli

Filippo Pietrantonio

Katja Pinker

Daniel Pipeleers

Miguel Ángel Piris Pinilla

Carlos Pirola

Bertram Pitt

Milan Piya

Mathias Pletz

Hans G Pohl

Stanislas Pol

Alberto Polimeni

David W Polly

Riccardo Polosa

Martí Pons-Òdena

Philip Poortmans

Thaddeus Pope

Craig Portell

Roos E Pouw

Paolo Pozzilli

Shyam Prabhakaran

Pranil Man Singh Pradhan

Robin Prescott

Jan Prins

Charlotte Probst

Spencer D Proctor

Christine Maria Prodinger

Tina Purnat

Margot Putukian

Lu Qi

Shanhu Qiu

Hua Qu

Jianchao Quan

Diego Quattrone

Gwendolyn Quinn

Miguel Quintela-Fandino

Luc Quintin

Franco Rabbia

Jack Rabi

Andrew Radley

Mohamed Rahouma

Armando Manuel Mendonça Raimundo

Kirsimarja Raitasalo

Noopur Raje

Paweł Rajwa

Karthik Ramamurthy

Sriram Ramgopal

Mao-Sheng Ran

Emanuel Raschi

Essam Rashed

James P Rathmell

Daphne Rattner

Vlad Ratziu

Vishnuga Raveendran

Eric Ravussin

Abdul Razak

Andrea Rehman

Erin Reilly

Paula Elaine Diniz Do Reis

Pryanka Relan

Xiubao Ren

Rungsun Rerknimitr

Ravi Retnakaran

Negar Rezaei

Taeho Greg Rhee

Jean Baptiste Ricco

Emma Ridley

Alan S Rigby

Lorenza Rimassa

Rosa Ritunnano

Nilo Riva

Ariadne Rivera-Aguirre

Eleni Riza

Alessandro Rizzo

Mimma Rizzo

Emily Roberts

Gregory William Roberts

Peter Robinson

Xavier Roblin

Marco Rocchi

Claire Roddie

Marcio L Rodrigues

Paula Rodriguez-Miguelez

Ana I Rodríguez-Pérez

Emiliano Rodriguez-Sánchez

Jane E Rogers

Kris Rogers

Claudia Roll

Renato Romagnoli

Annerine Roos

Norman Rose

Rafael Rosell

Stuart D Rosen

Simon Rosenbaum

Jacob Rosenberg

Joshua Rosenblat

Robert S Rosenson

David Ross

Jeanfrancois Rossi

Peter Rossing

Paisan Ruamviboonsuk

James Rudolph

Alice R Rumbold

Emma Russell

Even Rustad

Piotr Rutkowski

Harm Rutten

Ricardo Ruttimann

Miguel Angel Saavedra-Salinas

Maryam Saberi-Karimian

Carolyn Sachs

Marc Saez

Miia Sainio

Kirsten Salado-Rasmussen

Irving Salit

Jon Salmanton-García

Zainab Samaan

Jan-Stephan Sanders

Christina F Sandman

Carlo Sansone

Ponnusamy Saravanan

Amanda Veiga Sardeli

Kazunari Sasaki

Yasuharu Sato

Rob Saunders

Gregory Sawicki

Susan Sawyer

Lorenzo Scappaticcio

Matthew Schabath

Thomas Schenk

Steven Schiff

Alexandra Schifferli

Sandra Schlegl

Martin Schlumberger

Marcus Schmidt

Ricarda Schmidt

Ulrike Schmidt

Virginia A Schmied

Asbjørn Mathias Scholtens

Martin Schönlein

Benedikt Schoser

Christoph Schramm

Jörg Schulz

Giulia Scioscia

Thomas Scriba

Leonardo Sechi

Leonie Segal

Terry Segal

Jakob Benedict Seidelin

Anna Lene Seidler

David Seiffge

Isaac Seow-En

Aditi Shah

Amy Shah

Chirag Shah

Furqan Shaikh

Rory Shallis

Huifang Shang

Xuejun Shao

Yu-Yun Shao

Joshua Sharfstein

Kirthana Sharma

James Sharman

Oleg Shchelochkov

Aling Shen

Minxue Shen

Chen Shi

Meredith Shiels

Masayuki Shimoda

Samuel Shinjo

Anusha Shirwaikar Thomas

Kohei Shitara

Mercy Shobiye

Yehuda Shoenfeld

Lawrence Shulman

Lu Si

Spyridon Siafis

Margaret Sibley

Hafifa Siddiq

Márcio Roberto Silva

Robert Silver

Giovanni Antonio Silverii

Karen Simmer

David Simmons

Victoria Simms

Ana Cristina Simoes e Silva

Christian Simon

Alexander Singer

Mervyn Singer

Awadhesh Singh

Harminder Singh

Bireshwar Sinha

Bhawna Sirohi

Eline Smit

Dallas Smith

Kimberly R Smith

Jacqueline Sneddon

Yehoshua Socol

Annette Sohn

Maria José Soler Romeo

Francesca Solmi

Victor Søndergaard Wasehuus

Takuhiro Sonoyama

Teck Mun Soo

Sajid Soofi

Cynthia So-Osman

Kjetil Soreide

Vicente Soriano

Enrique Soto-Pérez-De-Celis

Sabri Soussi

Anna Maria Spagnolo

Francesco Spannella

Jean-Philippe Spano

Nicholas Spencer

Stavros C Spiliopoulos

Anne Spinewine

Matthew Spittal

Sandra Springer

Karen Spruyt

Derrick Ssewanyana

Jan Staessen

Don Staines

Joseph Standing

Sanja Stanojevic

Marina Stasenko

Ambra Stefani

Stephen Stefani

Armin Steffen

Coen Stehouwer

Jonathan Steinberg

Martin Steinhoff

Erik Stenberg

Kim Stene-Larsen

Lone Stensballe

Olof Stephansson

Terence Stephenson

Vicky Stergiopoulos

Alexandra Stevens

Gonneke W J M Stevens

Robert Stewart

Gregor Stiglic

Wilma Stolk

Jonathan Stone

Ole Jakob Storebø

Ragnhild Bjarkøy Strandberg

Pasquale Striano

Karien Stronks

Patrick Michael Stuart

Mohsan Subhani

Ashwin Subramaniam

Euisik Suh

David Sullivan

Jennifer Sumner

Bei Sun

Hsin-Yun Sun

Huichuan Sun

Li-Ying Sun

Louise Sun

Yehuan Sun

Yuhao Sun

Raghav Sundar

Clara Suñer

Riccardo A Superina

Jasjit Suri

Stefan Sutterlin

Gábor B Szabó

Fabio Silvio Taccone

Alphonse G Taghian

Heidi T Taipale

Mitsuyoshi Takahara

Kenzo Takahashi

Masahiro Takeshima

Hiroyoshi Takeuchi

Alison Talbert

Eng-King Tan

Ting Fang Tan

Tricia Tan

Atsushi Tanaka

Junko Tanaka

Ryota Tanaka

Tomotaka Tanaka

Weiming Tang

Weiwei Tang

Wenxi Tang

Pieter Tanis

Ian Tannock

Maxime Taquet

Lionel Tastet

Belaynew Taye

Matthew Taylor

Rod S Taylor

Jennifer Taylor-Cousar

Vannarath Te

Fabrizio Tediosi

Gao-Jun Teng

Weiping Teng

Michael L Terrin

Hendrik Tevaearai Stahel

Stephanie Than

Viengneesee Thao

Sudhin Thayyil

Linda Theron

Tinku Thomas

Sarah Thomis

Benjamin Thompson

Kelly Jane Thompson

Kimberly Thompson

Martin Thornhill

Allen Thornton

Dong Tian

Jie Tian

Phyllis Tien

Olena Tigova

Jari Tiihonen

Angela A Tincani

Andrea Tinelli

Darren Shu Jeng Ting

Svetlana Tishkovskaya

Nick Titov

Vivianne Tjan-Heijnen

Kelvin To

Louise Tofts

Masakazu Toi

Takahiro Tokunaga

Sara Tolaney

Janne Tolstrup

Bartłomiej Tomasik

Emily Tonorezos

Daniele Torella

Øivind Fredvik Grytten Torkildsen

Michelle Torok

Juan Torres-Macho

Hidenori Toyoda

Viet-Thi Tran

Suporn Treepongkaruna

Vladimir Trkulja

Tsen-Fang Tsai

Tung-Sung Tseng

Georgios Tsivgoulis

Yusuke Tsukamoto

Stefan Tullius

Natalie Uhlenbusch

Elizabeth Unger

Dipesh Uprety

Salvatore Vaccarella

Krishna Mohan Vadrevu

Luissa Vahedi

Louis J Vaickus

Alexander J C van Akkooi

Robert van Binnendijk

Inge van den Berk

Mark van den Boogaard

Henk van der Poel

Tori van Dyk

Ruben van Eijk

Wouter L W van Hemert

Bart van Hoek

Daniel van Hoving

Folkert van Kemenade

Zoé van kempen

Wilton A van Klei

Hanneke Van Laarhoven

Gert van Zyl

Nathan L Vanderford

Ilse Vanhorebeek

Yogesh K Vashist

Anca Vasiliu

Monica Vavilala

Kavita Vedhara

Anjo Veerman

Peter Ventevogel

Giovanni Vento

Sascha Verbruggen

Vicente Verez-Bencomo

Johan Verhaeghe

Geert Verleden

Suzanne Vernon

Christopher Verrico

Paul Verschure

Anne Vertigan

Jorma Viikari

Alessandro Villaschi

Antonella Vimercati

Annina Vischer

Antonio Vitiello

Deborah Vogt

Elizabeth Volkmann

Sebastian Vollmer

Charlotte Vrinten

Luan Vu

Alisha Wade

Nicholas Wald

David A Walker

Eric Yuk Fai Wan

Xiuhua Wan

Dan Wang

Duolao Wang

Jing Wang

Jing Wang

Jung-Der Wang

Kai Wang

Luowei Wang

Pu Wang

Wei Wang

Xiaohui Wang

Xin Wang

Yihua Wang

Yixin Wang

Yongjun Wang

Yu Wang

Zhao Wang

Saiama Waqar

Ahmed Waqas

Matthieu Wargny

Molla Wassie

Masayuki Watanabe

Nawat Watanachai

Emily Webb

Gwilym Webb

Ann Weber

Malte Wehmeyer

Yongyue Wei

Paul Weidle

Zvi Weizman

Jeremy Weleff

Mike Wells

Dirk Westermann

Jennifer Whitaker

Sarah White

Luuk Wieske

Annelies Wilder-Smith

Tanya Wildes

Emma Williams

Phoebe Williams

Gregory Willis

Calistus Wilunda

Pattanee Winichagoon

Peter Winocour

Bettina Winzeler

Grace Wong

Mark Woodward

Gillian Worthy

Charlotte Wright

Chih-Hsing Wu

Chongming Wu

Depei Wu

Hongjiang Wu

Hsien-Tsai Wu

Huangan Wu

Joy Tzung Yu Wu

Pingsheng Wu

Robert Wu

Shishi Wu

Yongda Wu

Alysse Wurcel

Zhengyuan Xia

Shangmin Xiong

Shangzhi Xiong

Lan-Ping Xu

Miao Xu

Pan Xu

Xianghuai Xu

Zhenghao Xu

Lalit Yadav

Ravi Yadav

Martin Yaffe

Koray Yalcin

Hideto Yamada

Lijing Yan

Lu Yang

Minghui Yang

Rui Yang

Robert C Yarchoan

Byong Duk Ye

Weimin Ye

Xin Ye

Chihjung Yeh

Musa Yilmaz

Peng Yin

Guishuang Ying

Terry Yip

Tomoya Yokota

Kate Young

Ilan Youngster

Chiahung Yu

Honghua Yu

Jin-Tai Yu

Ming-Lung Yu

Yunfang Yu

Ying Yuan

Zhiyong Yuan

Courtney Yuen

Sophia Zackrisson

Elena Zapata-Arriaza

Dorota Zarębska-Michaluk

Laurie Zawertailo

Johan Zelano

Hongmei Zeng

Fengqing Zhang

Hai-Jun Zhang

Heng Zhang

Hongxia Zhang

Hua Zhang

Hui Zhang

Huimin Zhang

Jingyao Zhang

Jintao Zhang

Lei Zhang

Li Zhang

Meng Zhang

Peng Zhang

Wencheng Zhang

Wenwen Zhang

Xiulan Zhang

Yan-Bo Zhang

Zhe Zhang

Bin Zhao

Binghao Zhao

Fengmin Zhao

Joseph Zhao

Shihua Zhao

Shuang Zhao

Yihong Zhao

Yuanjin Zhao

Lai-ping Zhong

Lianzhen Zhong

Jian-Xin Zhou

Jiawei Zhou

Yi-Hua Zhou

Yujie Zhou

Beibei Zhu

Feng-Cai Zhu

Guanghu Zhu

Qingli Zhu

Taiyu Zhu

Xiao-Li Zhu

Zhuoting Zhu

Chengle Zhuang

Hongqing Zhuang

Hana Zickgraf

Focke Ziemssen

Eduardo Zimmer

Vincent Zimmer

Stephan Zipfel

Andres Zorrilla-Vaca

Paolo Zucali

Gianvincenzo Zuccotti

Marco Zuin

Garazi Zulaika

Andrea Zülke

Christian Zwaan

Sa’ed H Zyoud

